# The Prevalence of Pediatric Lower Limb Fractures Following Motor Vehicle Accidents at King Abdullah Specialist Children's Hospital, Riyadh, Saudi Arabia

**DOI:** 10.7759/cureus.28724

**Published:** 2022-09-03

**Authors:** Abdulmajeed S Almansouf, Omar Khalid Alkhanbashi, Sultan Alsumairi, Naif A Alhussein, Meshari Alosaimi, Abdulaziz A Alquraishi, Amal Yousif

**Affiliations:** 1 Medicine, King Saud bin Abdulaziz University for Health Sciences, College of Medicine, Riyadh, SAU; 2 Emergency Medicine, King Saud bin Abdulaziz University for Health Sciences, College of Medicine, Riyadh, SAU; 3 Pediatric Emergency, Princes Nora University, Riyadh, SAU

**Keywords:** trauma, pediatrics, motor vehicle accidents, lower extremity, fractures

## Abstract

Background

Pediatric injuries are a very common and serious issue that may result in permanent harm. Regardless of the etiology of the injury, children represent up to 25% of all cases visiting the emergency room. Children suffer nearly twice as many fractures as adults, with males being more vulnerable to this type of injury.

Methods

A retrospective cohort study was conducted among 2777 patients, and a total of 203 patients who fit the inclusion criteria were included. Data were collected using the electronic system at King Abdullah Specialist Children's Hospital (KASCH). Participants were selected by a non-probability purposive sampling method and data analysis was carried out using the SPSS program.

Results

The prevalence of motor vehicle accidents (MVAs) related to lower limb fractures was estimated to be 7.3%. The patients had an average mean age of 8.6±4 years, with 10-14 years being the most common age group affected (48.3%) with male predominance (72.4%). Furthermore, femoral fractures were the most commonly identified type (31.5%), and a pedestrian was the most common mechanism of multiple lower limb fractures. Additionally, the highest reported mechanism of injury of lower limb fractures was MVAs. Moreover, the current study shows that most of those patients were treated surgically (55.1%).

Conclusion

Conclusively, our cohort estimated that the prevalence is 7.3% of MVAs-related lower limb fractures among all children presented to the KASCH Emergency Department. Our study showed that children who were 10-14 years of age were the most affected. There is a male over female predominance. A femur was the most common bone to be affected. Lastly, further safety awareness programs and campaigns are important to be initiated by governmental authorities.

## Introduction

Bone fracture is defined as a medical condition in which the bone's continuity is broken either partially or completely [1]. Fractures are common in all forms of pediatric injuries, accounting for 10-25% of all cases [2]. Children's fractures are almost twice the number of adults, with the male gender being at higher risk [2,3]. Fractures can result from multiple injury mechanisms such as twisting injuries, falls either from above or below bed height (<1 m), falling down the stairs or slopes, blunt trauma, sports injuries, and traffic accidents [3]. A study that was conducted in China pointed out that MVAs (47%) and low falls (26%) followed by high falls (15%) then struck by an item (4.4%) were the most common causes of injury among children of all age groups and both genders [4]. Moreover, a study conducted in the USA reported that two-thirds of the injuries were caused by MVAs and falls, with falls being more common in younger children and MVAs being more common in older children [5].

MVAs are the world's eighth leading cause of mortality, especially among the young population. Every year, around 1.35 million die and millions more are injured and admitted to hospitals [6]. Although the exact number of children injured or disabled each year because of MVAs worldwide is unknown, the World Health Organization estimates that it is around ten million. In Saudi Arabia, the bulk of all cases admitted for fractures as well as trauma treated at the emergency departments are caused by traffic accidents, followed by falls [7,8]. Age-wise, children are at a higher risk for pedestrian injuries as their biopsychosocial characteristics, such as gross motor, cognitive, perceptual, judgmental, and social skills, all affect their capacity to respond successfully to traffic hazards [9]. A Singapore study supports this claim as their research showed that most pediatric pedestrian injuries involved primary schoolers returning home from school unaccompanied by adults [10]. A review of MVAs in the Arabian Gulf countries revealed that Saudi Arabia had the highest incidence of pedestrian accidents, and the majority of children presented with lower limb injuries were due to this type of accident [9].

Mechanisms of injury and location of fracture have an evident correlation, as studies reported that sports injuries and falls more commonly result in upper limb fractures, whereas MVAs tend to cause lower limb fractures [11]. Lower limb fractures as a result of MVAs can lead to significant morbidity and are responsible for a huge part of nonfatal injuries requiring hospitalization, accounting for up to 20% of all admissions [12]. A study in China reported that the most common fracture sites were lower extremity (60.0%) and craniofacial (30.8%), followed by upper extremity (17.0%) [2]. Furthermore, a study in Saudi Arabia showed that out of 217 children with lower extremity fractures, 141 had femur fractures, 47 with tibia fractures, and 50 with pelvic fractures [9]. Recent literature in Saudi Arabia supports this claim and reported that the femur (25.9%) is the most commonly fractured bone [1]. In addition, not only are fractures the most common consequence of MVAs, but also lower limb fractures may lead to functional limitation one year following hospital discharge [12]. In conclusion, parents and hospitals alike are concerned about fractures and dislocations in growing children because any mismanagement results in a permanent impairment and a significant socio-economic burden on the country [13]. There are few studies and limited data about lower limb fractures, particularly in Saudi Arabia. The aim of this study is to enhance the epidemiological view of this topic within the Kingdom of Saudi Arabia by using the patients' data visiting the emergency department of King Abdullah Specialist Children's Hospital (KASCH) in order to estimate the prevalence of pediatric lower limb fractures following MVAs.

## Materials and methods

Study area and design

This study was conducted at the emergency department of KASCH, Riyadh, Saudi Arabia. The hospital offers multiple services in many specialties, from outpatient clinics to fully equipped diagnostic and therapeutic inpatient wards. KASCH has around 550 beds in total for inpatient care and 60 beds for the emergency department and trauma. The study design used was a retrospective cohort chart review. 

Inclusion and exclusion criteria

The population of the inclusion criteria included all patients under the age of 14 who had traumatic fractures due to MVAs from January 1, 2016, to January 1, 2021. Patients who were dead at the scene or upon arrival at the emergency department and those who suffered from chronic illnesses known to make the bones more vulnerable to fractures were excluded, such as osteogenesis imperfecta, vitamin D disorders, hypophosphatasia, infantile osteoporosis, etc.

Data collection

Data collection, which was done by the research team only, was through the hospital's Best-Care digital medical records system. The data were collected from January 1, 2016, to January 1, 2021, using a retrospective chart review by going through all patients' electronic medical files.

Statical analysis

The program used for data analysis was SPSS (IBM, Inc., Armonk, USA), utilizing descriptive and categorical data. Multivariate logistic regression analysis was used to estimate adjusted odds ratio (AOR) and 95% confidence intervals (CIs) for sample differences. The test was considered significant if the p-value was less than 0.05.

## Results

Between January 1, 2016, to January 1, 2021, a total of 2777 pediatric patients (aged 0-14 years) who sustained traumatic lower limb fractures were presented to the emergency department. Two hundred and three, representing 7.3% of total patients, had lower limb fractures following MVAs. The other 2574 (92.7%) suffered from fractures due to other causes such as falling either from >1 m or below, twisting injuries, crushing fractures due to heavy objects, or other causes. The patients had an average mean age of 8.6±4 years, with 10-14 years being the most common age group affected (48.3%) with male predominance (72.4%). In terms of fractures type, femoral fractures were the most commonly identified type (31.5%), which was mostly due to car accidents with singular rather than multiple lower limb fractures. On the other hand, pedestrian accidents were the most common mechanism of multiple lower limb fractures. Moreover, most of the patients included in this study were treated surgically (55.1%).

Patients characteristics

The current study indicates that patients aged 10-14 years old had the highest prevalence of lower limb fractures due to MVAs (n=98, 48.3%), followed by 4-9 years (n=79, 38.9%). Most of the patients were males (n=147, 72.4%) while females were 27.6% (n=56). The patients had an average mean age of 8.6±4 years old with mean weight, height, and BMI of 34.7±20.5, 130.6±26.2, and 18.5± 5.3, respectively (Table 1).

**Table 1:** Socio-demographic characteristics of pediatric patients (≤14 years) who underwent traumatic fracture

Age groups	n (%)
0 - 3	26 (12.8)
4 - 9	79 (38.9)
10 - 14	98 (48.3)
Gender	n (%)
Male	147 (72.4)
Female	56 (27.6)
Weight: mean (SD)	34.7±20.5
Height: mean (SD)	130.6±26.2
Body mass index: mean (SD)	18.5±5.3

Type of fractures

**Figure 1:**
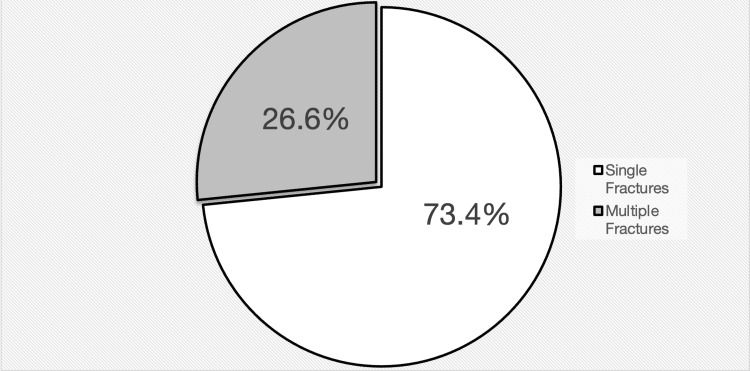
Distribution of traumatic lower limb fractures among pediatric patients (≤14 years)

Among 2777 pediatric patients, a total of 203 (7.3%) patients who fit the inclusion criteria had 267 lower limb fractured bones, most of which had singular (73.4%) rather than multiple ipsilateral and contralateral (26.6%) lower limb fractures (Figure 1). Among the lower limb fractures, femoral fractures were the most commonly identified type (n=85, 31.5%), most commonly due to car accidents (n=58). This is followed by tibial fractures (n=80, 30%) and fibular fractures (n=37, 13.9%), mostly suffered by pedestrians. Moreover, pubic fractures constitute 6.4% (n=17), distributed equally between cars and pedestrian accidents, while metatarsal and phalangeal fractures constitute 6% (n=16) and 8,3%, respectively, mostly caused by motorcycle accidents (Table 2).

**Table 2:** Type of lower limb bone fractures and their mechanisms among pediatric patients (≤ 14 years) who underwent treatment

Type of lower limb fractures	n (%)	Car	Pedestrian	Motorcycle
Pubis	17 (6.4)	8	9	0
Acetabulum	5 (1.8)	4	1	0
Sacral ala	1 (0.4)	1	0	0
Iliac bone	2 (0.8)	1	1	0
Femur	84 (31.5)	58	20	6
Patella	1 (0.4)	0	0	1
Tibia	80 (29.9)	31	36	11
Fibula	37 (13.9)	11	20	6
Ankle	3 (1.1)	3	0	0
Malleolus	5 (1.8)	2	1	2
Cuneiform bone	2 (0.8)	0	2	0
Calcaneus	2 (0.8)	1	0	1
Navicular bone	2 (0.8)	0	1	1
metatarsals	16 (5.9)	1	5	10
Body of symphysis	1 (0.4)	1	0	0
Phalanx	8 (2.9)	0	1	7
Pelvis	1 (0.4)	1	0	0
Total	267 (100%)	123	97	45

Mechanisms of fractures

**Figure 2:**
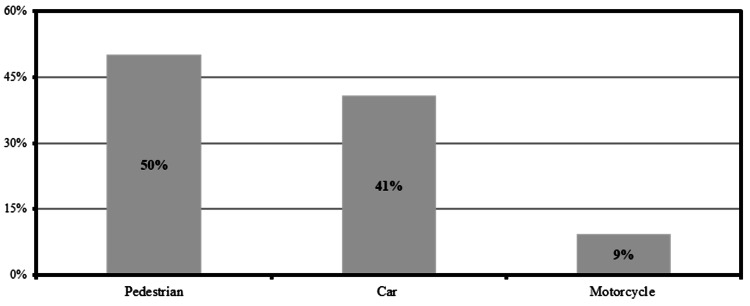
Mechanisms of injury causing traumatic multiple lower limb fractures among pediatric patients (≤14 years)

Table 2 lists the most commonly reported mechanisms of injury of lower limb fractures. The table indicates that car accidents constitute 46% (n=123), followed by pedestrians (n=99, 37%), and motorcycle (n=45, 16.8%). However, half of the multiple lower limb fractures were sustained by pedestrians, followed by car accidents (41%) and motorcycle accidents (9%; Figure 2). The femur was the most common bone fractured by car accidents (47.2%), whereas the tibia was the most common fractured bone due to pedestrians (37.1%) and motorcycle accidents (24.4%; Table 2).

**Figure 3:**
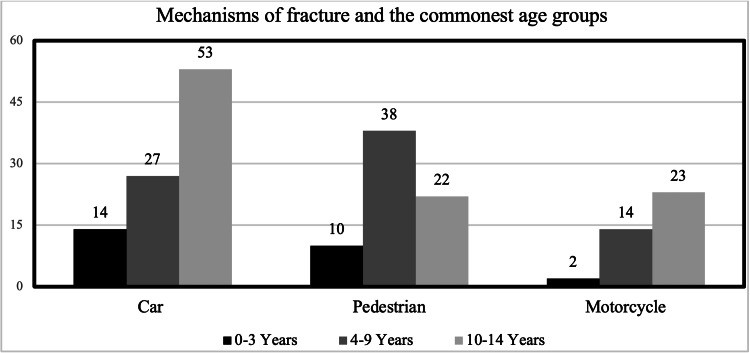
Age groups that sustained traumatic lower limb fractures via different mechanisms among pediatric patients (≤14 years)

The most common age groups that sustained lower limb fractures by each mechanism are presented in Figure 3. Car accidents were the main mechanism of injury for the 10-14 years age group with 53 incidents, and the 4-9 years age group had the pedestrians with highest incidents with 38 cases, while the motorcycle was the main cause for accidents in 10-14 years age group with 23 cases. Table 3 indicates the number of patients with multiple lower limb fractures distributed by age group and injury mechanisms. The data indicates that the 10-14 years age group was mostly affected by car accidents (n=16), whereas the 4-9 years age group was mostly subjected to multiple lower limb fractures due to pedestrian accidents (n=18) and motorcycle accidents (n= 3). These relationships were found to be statistically significant (p=0.004).

**Table 3:** Age groups with traumatic multiple lower limb fractures via different mechanisms among pediatric patients (≤14 years)

Mechanism of Injury	Age group (years)	Number (%)
Car	0 - 3	1 (4.6)
	4 - 9	5 (22.7)
	10 - 14	16 (72.7)
Pedestrian	0 - 3	2 (7.4)
	4 - 9	18 (66.7)
	10 - 14	7 (25.9)
Motorcycle	0 - 3	0 (0)
	4 - 9	3 (60)
	10 - 14	2 (40)

Management of the fractures and outcomes

Surgical management was the most common modality used to treat fractures (55.1%), followed by conservative management. Patients aged 0-3 and 4-9 years were mostly managed conservatively (61.5% and 50.6%, respectively). Patients aged 10-14 years were managed surgically (64.3%), and these relationships were found to be statistically significant (p=0.026).

Most of the patients of car accidents were managed in the operation room (69.1%), whereas fractures that occurred to pedestrians and those caused by motorcycle accidents were managed conservatively (55.7% and 59%, respectively). These associations were statistically significant (p=0.001). Table 4 shows details of the types of management, indicating that the 10-14 age group is more prone to accidents with the highest surgical management involvement. On the contrary, the 0-3 age group was the least group subjected to accidents and required less need to surgical intervention. Overall, within the cases considered, there were more those who needed surgery than those who were treated conservatively (n=112 vs. n=91).

**Table 4:** Type of management among different age groups and types of injuries among pediatric patients (≤14 years) with traumatic fractures

Age groups	Conservative	Surgical	p-value
0 - 3	16 (61.5)	10 (38.5)	0.026
4 - 9	40 (50.6)	39 (49.4)	
10 - 14	35 (35.7)	63 (64.3)	
Mechanism of injury	Conservative	Surgical	p-value
Car accidents	29 (30.9)	65 (69.1)	0.001
Pedestrian	39 (55.7)	31 (44.3)	
Motorcycle accidents	23 (59)	16 (41)	

**Figure 4:**
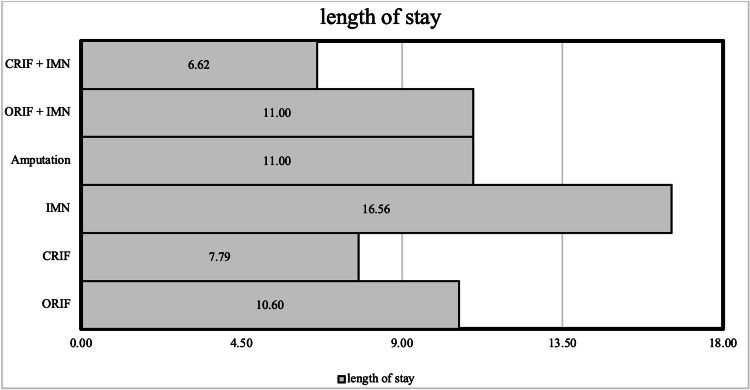
Duration of hospital stay of different types of surgical operations among pediatric patients (≤14 years) with traumatic fractures CRIF+IMN - closed reduction internal fixation with intramedullary nailing; ORIF+IMN - open reduction internal fixation with intramedullary nailing; IMN - intramedullary nailing; CRIF - closed reduction internal fixation; ORIF - open reduction internal fixation

Considering the length of stay, patients who suffered car accidents had the longest duration, with a mean stay duration of 11.31 days due to the necessity of an open and more extensive surgical intervention, whereas pedestrian and motorcycle incidents have a mean duration of nine and seven days, respectively. Figure 4 indicates the duration of hospital stay for different types of surgical operations. The figure indicates that closed surgeries, including closed reduction internal fixation with intramedullary nailing (CRIF+IMN) and closed reduction internal fixation (CRIF), were associated with the shortest stay with a duration of 6.62 and 7.79 days, respectively. Open surgeries, including open reduction internal fixation with intramedullary nailing (ORIF+IMN), open reduction internal fixation (ORIF), amputation, and intramedullary nailing (IMN), required longer stay with 11.0, 10.6, 11.0 days for the first three surgeries, respectively, with the longest duration of stay of 16.56 days for IMN.

## Discussion

MVAs are the eighth leading cause of morbidity and mortality for millions of people around the world, especially in children [6]. Furthermore, lower limb fractures account for approximately 20% of all fractures in children and may result in substantial mortality and morbidity [14,15]. Both health policy-makers and healthcare providers can benefit from the epidemiological studies of MVAs injuries. Healthcare providers can benefit from the information on lower limb fractures and their possibility of being multiple and complicated based on patients' age and/or the mechanism of injury to improve the assessment and treatment of patients and help make the clinical decision. Health policy-makers can utilize injury patterns and epidemiological data to form legislation and prevention measures and allocate resources. In Saudi Arabia, the bulk of all cases admitted for fractures as well as trauma are caused by traffic accidents, followed by falls [8]. Some studies have shown the incidence of specific lower limb fracture sites and specific etiology in pediatric patients, but data on the overall patterns and epidemiologic trends have not been covered extensively in Saudi Arabia [16,17]. The current study investigated the epidemiology of lower limb fractures among children who presented at the emergency department of KASCH, Riyadh, Saudi Arabia.

Accidents constitute a major cause of morbidity and mortality in childhood, and studies have explained the increase in fractures rate during the pubertal years and related that to height gain and accrual of bone mineralization [18]. Furthermore, surveys of pediatric trauma have suggested that fractures contribute to 10-25% of all injuries, with males being more susceptible [19,20]. In this study, a total of 2777 pediatric patients aged 0-14 years presented at KASCH with lower limb fractures. Out of the total number, 203, representing 7.3% of the total number of patients, had lower limb fractures following MVAs. The current data confirm that there is considerable variation in the prevalence of lower limb fractures by age and sex. Also, the occurrence of fractures increased as age increased, with the 10-14 years age group representing the largest proportion of the patients accounting for 48.3%. This is logical as this age group is more associated with mobilization and physical activity, hence prone to accidents. In terms of gender, males are predominant, which is in line with other worldwide reports [7-11,17,18]. The difference in gender and age groups regarding the incidence of fractures is well-known.

Car accidents represent the most commonly reported mechanism of injury of lower limb fractures (n=123, 46%), most of which were treated surgically. This was consistent with other studies indicating that surgery is the most common treatment modality for such cases [8,12,13]. According to the current data, the most common fractured bone was the femur representing 31.5%, mainly due to car accidents, followed by tibia fractures that occurred mainly in pedestrians, which constituted 29.9% of all fractures. Previous studies indicate that the tibial shaft was the most common fracture site in the lower limb region [21,22]. However, recent literature in Saudi Arabia supports the current study's findings by reporting the femur as the most commonly fractured bone representing 25.9% of all fractures [1].

In terms of treatment modality, the current findings show that most children who sustained femoral fractures were managed and treated surgically (55.1%), and this agrees well with the findings of the previous study [12]. On the contrary, a study done in Qatar indicates that the majority of children who sustained such a fracture had been treated conservatively [23]. The current study points out that fractures related to car accidents are mostly managed surgically and require the longest duration of hospital stay with a mean of 11.31 days. This is explained by the fact that car accidents are high-energy trauma that often leads to open fractures, multiple soft tissue injuries, and other complications that pose a serious threat to children's health and often require lengthy hospitalization. Therefore, parents, schools, community, and authorities should play their role in promoting safety precautions in general and traffic safety in specific, including being cautious crossing streets, using car safety seats, and wearing protective gear while riding bicycles and motorcycles, all with increasing supervision.

Finally, it is worth mentioning that this work is a hospital-based cross-sectional study; hence, the generalizability is limited, and the causality cannot be identified and evaluated. Therefore, further multicentered studies with advanced designs are needed.

## Conclusions

This work presents a hospital-based epidemiological study of children's fractures at KASCH. Our results suggest that such fractures are a common problem and showed that children 10-14 years of age were the most affected, with evident male predominance. The femur, followed by the tibia, were the most common bones to be affected, and car accidents were the most common mechanism of injury. In general, surgical management was the most practiced treatment modality, and such management was practiced extensively in the 10-14 years age group and mostly in car accident patients. Open surgeries were associated with a longer duration of hospital stay, and patients of car accidents who had surgery required the longest hospital stay. Based on these findings, a multidisciplinary approach is needed to minimize car accidents in general and specifically in the pediatric population. Traffic and safety education programs and community campaigns should be carried out in intervention with schools. More road signs, clear pedestrian crossings, and adult supervision are essential to maintain the children's safety. Moreover, strict rules must be applied by authorities to cut down on these types of accidents.
